# New Predictive Diagnostic Method for Cardiac Dynamics Based on Probability Distributions

**DOI:** 10.3390/diagnostics15060650

**Published:** 2025-03-07

**Authors:** Javier Rodríguez Velásquez, Leonardo Juan Ramírez López, Sofia García Torres

**Affiliations:** 1 Harmonyk Research Group, Bogota 110861569, Colombia; 2 TIGUM Research Group, Universidad Militar Nueva Granada, Bogotá 110111481, Colombia

**Keywords:** probability, distributions, cardiac, anomalies, diagnostic, critical care

## Abstract

**Background/Objectives:** Probability theory and dynamic systems have enabled the development of diagnostic support tools that simplify Holter evaluation. **Method:** A study was conducted on 80 Holter tests over 21 h with patients over 21 years old. Four prototypes were selected based on normal, chronic, acute, and pacemaker diagnoses. An induction was created using the heart rate ranges of the prototypes, from 55 to 105, as the general probability space. Probability theory was applied to the frequency repetition ranges of 1000 to 2000 and 2001 to 3000. A blinded study was conducted with the remaining Holter tests, applying the same methodology used for the prototypes. A physical/mathematical induction was performed for the prototypes, and the other Holter tests were analyzed in a blinded study. **Results:** The results were compared to the predictions of the prototypes, and sensitivity, specificity, and the kappa coefficient were calculated. In the 1000–2000 range, the repetition counts for normal dynamics were 14 to 11, for chronic cases 31 to 21, for acute cases 11 to 9, and for pacemaker dynamics 5 to 4. In the 2001–3000 range, the repetitions for normal dynamics were 3 to 0, for chronic cases 14 to 10, for acute cases 6 to 3, and for pacemaker dynamics 2. The cumulative probabilities loaded for the 1000–2000 range were as follows: normal dynamics, 0.46 to 0.35; chronic dynamics, 0.48 to 0.35; acute cases, 0.6 to 0.5; and pacemaker dynamics, 0.6 to 0.5. In the 2001–3000 range, the cumulative probabilities loaded for normal dynamics were 1 to 0; for chronic cases, 0.7 to 0.54; for acute cases, 0.75 to 0.46; and for pacemaker dynamics, 1. The frequencies observed in the repetition ranges for 1000–2000 were normal, 95 to 55; chronic, 105 to 65; acute, 100 to 75; and pacemaker, 75 to 60. For the 2001–3000 range, the frequencies were normal, 95 to 65; chronic, 85 to 65; acute, 100 to 80; and pacemaker, 65 to 60. The probabilities were less than 0.3 for normal dynamics and greater than 0.3 for chronic, acute, and pacemaker dynamics across different frequency ranges, differentiating the dynamics. **Conclusions:** The epidemiological study results for sensitivity, specificity, and kappa coefficient were all 1. To conclude, a diagnostic support tool was developed for cardiac dynamics with clinical applications based on the appearance of frequency ranges and probability theory, enabling differentiation of normal, chronic, acute, and pacemaker dynamics.

## 1. Introduction

The quantification of the likelihood of a potential future event within a totality of events is calculated using probability theory [1,2]. This results in a dimensionless mathematical measure ranging between 0 and 1, as shown in [3], calculated through an event space [4]. The application of probability theory has enabled the development of new methodologies for cardiac dynamics, establishing parameters for studying state and evolution. This allows differentiation between various types of dynamics [5]. This methodology has also been applied to the cardiac dynamics of patients with pacemakers, analyzing the state and progression of these dynamics [6]. Additionally, it has been used to analyze patients with arrhythmias, where parameters were applied to characterize the behavior of these dynamics [7].

In modern physics, theories are both acausal and predictive, as seen in relativity and quantum mechanics [8,9,10]. In the development of dynamic systems theory, chaos theory has emerged, studying how a system evolves through chaotic attractors. This theory has been applied to cardiology [11]. Rodríguez et al. developed a methodology using the Box-counting general space [12], successfully distinguishing normal and acute dynamics through geometric occupancy spaces.

Goldberger introduced a health/disease concept based on the application of dynamic systems to cardiac physiology. He demonstrated that highly regular or highly irregular dynamics indicate signs of disease, while dynamics lying between these extremes indicate a state of normality [11]. Other physical and mathematical methodologies have been developed based on chaos theory and the concept of a “loaded die”, creating cardiac dynamics diagnostics using an exponential law applied to Holter tests [13]. Probability theory has also been applied to patients with arrhythmias, enabling the identification of underdiagnosed conditions [14,15]. Another study used entropy-based methodologies to monitor fetal dynamics, assessing the state and progression of fetal tracings [16]. These methodologies are grounded in an event-loading concept and probabilities developed by Rodríguez referred to as the “loaded die principle”. This principle posits that determinism and indeterminism coexist, suggesting that the world is neither completely deterministic nor entirely indeterministic but a combination of both [17].

New methodologies have also been developed for Holter evaluation. One such method is Zipf’s law, which originated from the study of natural languages. Later, Benoit Mandelbrot extended it to calculate complexity levels in this law. Analogies between language and the immune system’s functioning in T and B cell repertoires have been drawn [18,19], with clinical applications involving allergens such as Poap9 [20].

In the same way, a hierarchical order of hourly heart rate frequencies has been observed, showing hyperbolic behavior analogous to natural languages, as previously applied to the immune system, and demonstrating statistical fractal self-organization. Through an induction process followed by a blinded study with 20 Holter recordings, the complexity degree range was found to be between 0.7046 and 0.9483 for normal dynamics and between 0.428 and 0.6707 for acute dynamics. The clinical validation results showed 100% sensitivity, specificity, and kappa coefficient [21]. Other studies have also calculated the complexity degrees of cardiac dynamics for clinical applications [22,23].

This methodology has also been applied to fetal cardiac dynamics, developing a diagnostic support tool for fetal monitoring. It calculates the statistical fractal complexity degree based on the emergence of dynamic system components, examining different base and height areas of fetal cardiac dynamics. These dynamics showed hyperbolic behavior analogous to natural languages. The complexity degree for each monitoring session was determined, with normal fetal monitor readings displaying higher complexity degrees than those of pathological cases [24].

Globally, 16% of deaths are attributed to cardiovascular diseases (CVD), with an exponential increase in mortality rates. In 2019, CVD caused over 9 million deaths worldwide, making it the leading cause of death [25].

The Holter test records cardiac dynamic variability, storing it in an electronic channel to analyze R-R interval variability, minor rhythm changes, and cardiac rhythm alterations. This non-invasive test requires a minimum recording period of 21 h [26]. Additionally, the Holter device can examine the rhythm and state of patients with pacemakers, recording daily cycles, retention anomalies, and pacemaker capture and output performance [27]. The pacemaker is implanted in patients requiring assistance for proper heart function, providing electrical impulses to facilitate contraction or relaxation during blockages or irregularities in the heart’s electrical activity. Depending on the heart’s condition, the device activates specific functionalities, delivering consistent electrical contractions to maintain cardiac performance [28]. This device has enabled better management of cardiac diseases that pharmacological treatments alone cannot address, extending life expectancy for patients with stroke [29].

This study aims to develop a diagnostic support tool to predict the behavior of cardiac dynamics.

## 2. Materials and Methods

A total of 80 Holter tests recorded over 21 h were analyzed, involving patients aged over 21 years. From these, 4 prototypes were selected, representing normal, chronic, acute, and pacemaker diagnoses.

Normal, chronic, acute, and pacemaker dynamics are selected based on the gold standard for Holter evaluation, assessed by cardiologists. These dynamics are entirely distinct from one another, with no diagnostic uncertainty, allowing for a generalization to other possible dynamics. The frequency distributions are completely different, and by applying probability theory, these differences are quantified.

The remaining tests included 16 normal, 20 chronic, 20 acute, and 20 pacemaker cases, with diagnoses confirmed through clinical consensus. These studies were sourced from the Harmony research group’s Holter database (Bogota, Colombia), examined by a specialist, and adhered to parameters established by the gold standard (Table 1).

A physical/mathematical induction was created by taking the maximum, minimum, and intermediate heart rates from the prototypes, grouped into 5-beat-per-minute ranges. These ranges were placed into a general probability space and divided into two repetition ranges: 1000 to 2000 and 2001 to 3000. The equally probable probability for each range was calculated relative to the total repetition ranges within the probability space. The most frequent probabilities for each repetition range were then summed.

### Statistical Analysis

Finally, the heart rates from the remaining 76 Holter recordings were analyzed while concealing the diagnoses, conducting a blind study. The same probability methodology applied to the prototypes was then used for the repetition ranges of 1000 to 2000 and 2001 to 3000. The probability methodology was applied to the prototypes, and then the diagnoses were revealed to calculate sensitivity, specificity, and the kappa coefficient. The kappa coefficient measurements are performed for each dynamic in comparison with the gold standard.

The materials used in this research complied with the scientific, technical, and administrative standards for health research, as established by Resolution No. 008430 of 1993 from the Ministry of Health, Article No. 11, which outlines regulations for risk-free studies in human subjects. Since the study analyzed results from medical tests without directly intervening with patients or using personal information, it posed no risk to participants.

Some definitions:

Heart rate repetitions: The number of times a specific heart rate frequency is repeated during a Holter test.

Probability theory: A mathematical framework that quantifies the likelihood of an event occurring, expressed as a real numerical value between 0 and 1.

## 3. Results

Overall repetitions: Across all dynamics, the repetitions in the 1000–3000 range varied from 31 to 0. The sum of the most frequent probabilities in these ranges also ranged from 1 to 0. Frequencies where these probabilities appeared were between 105 and 55 bpm.

Repetition distribution: For normal dynamics, repetitions in the 1000–3000 range were between 14 and 0, chronic dynamics ranged from 31 to 10, acute dynamics from 11 to 3, and pacemaker dynamics from 5 to 2. Within the 1000–2000 range, normal repetitions were between 14 and 11, chronic repetitions between 31 and 21, acute repetitions between 11 and 9, and pacemaker repetitions between 5 and 4, see Table 2.

Number of times heart rates repeated within the ranges of P1 1000 to 2000 in normal, chronic, acute, and pacemaker prototypes.

- FREQ: Heart rate recorded by the test according to the repetitions within the ranges;
- PROT: Prototype;
- REP: Number of repetitions observed during the Holter test.

Within the 2001–3000 range, normal repetitions ranged from 3 to 0, chronic repetitions from 14 to 10, acute repetitions from 6 to 3, and pacemaker repetitions stayed at 2 (Table 3).

Number of times heart rates repeated within the ranges of P2 2001 to 3000 in normal, chronic, acute, and pacemaker prototypes.

- REP: Number of repetitions observed during the Holter test;
- FREQ: Heart rate recorded by the test according to the repetitions within the ranges.

Sum of probabilities: For the 1000–3000 range, the sum of the most frequent probabilities was as follows: normal dynamics: 1 to 0; chronic dynamics: 0.7 to 0.35; acute dynamics: 0.75 to 0.46; pacemaker dynamics: 1 to 0.5. For the 1000–2000 range, normal dynamics: 0.46 to 0.35; chronic dynamics: 0.48 to 0.35; acute dynamics: 0.6 to 0.5; pacemaker dynamics: 0.6 to 0.5. For the 2001–3000 range, normal dynamics: 1 to 0; chronic dynamics: 0.7 to 0.54; acute dynamics: 0.75 to 0.46; pacemaker dynamics: 1 (Table 4).

Summation of the most frequent probabilities in the ranges P1 (1000 to 2000) and P2 (2001 to 3000) for normal, chronic, acute, and pacemaker dynamics.

- DX: Diagnosis;
- MAXIMUM: The highest result among all summations;
- MINIMUM: The lowest result among all summations.

Frequency distributions: The 1000–3000 range: normal frequencies between 95 and 55 bpm; chronic frequencies between 105 and 65 bpm; acute frequencies between 100 and 75 bpm; pacemaker frequencies between 75 and 60 bpm. The 1000–2000 range: normal: 95 to 55 bpm; chronic: 105 to 65 bpm; acute: 100 to 75 bpm; pacemaker: 75 to 60 bpm. The 2001–3000 range: normal: 95 to 65 bpm; chronic: 85 to 65 bpm; acute: 100 to 80 bpm; pacemaker: 65 to 60 bpm. Probability distributions were always <0.3 for normal dynamics and >0.3 for chronic, acute, and pacemaker dynamics. Chronic dynamics showed a probability of 0.3 between 75 and 85 bpm. Acute dynamics reached 0.5 at 90 bpm. Pacemaker dynamics displayed a probability of 0.5 at 60–65 bpm. These distributions and frequencies are highlighted, differentiating the dynamics (Table 5). As to the epidemiological analysis, the calculations showed sensitivity, specificity, and kappa coefficient results of 1 in all cases.

Number of repetitions within the ranges of P1 (1000 to 2000) and P2 (2001 to 3000) observed in the heart rates of the prototypes: normal, chronic, acute, and pacemaker.

- FREQ: Heart rate frequency;
- RF: Number of times the repetition occurred in the heart rate;
- PROB: Probability of the number of times a repetition occurred in the frequency relative to the total repetitions;
- PROT: Prototype.

## 4. Discussion

A blind study confirmed the ability to differentiate one cardiac dynamic from another, showing that, in the repetition ranges, probabilities ranged from 1 to 0 in normal cases, from 0.7 to 0.35 in chronic cases, from 0.75 to 0.46 in acute cases, and remained at 1 in pacemaker cases. These probabilities occurred at specific frequency ranges: 95–55 for normal cases, 105–65 for chronic cases, 100–75 for acute cases, and 75–60 for pacemaker cases. The dynamics were distinguished by different ranges of frequencies for loaded probabilities, which were below 0.3 for normal cases and above 0.3 for chronic, acute, and pacemaker cases. Results from the blind study showed a concordance of 1 in sensitivity, specificity, and kappa, confirming that the physical and mathematical discriminations are precise and objective. Using this methodology, a diagnostic tool was developed that can differentiate all types of cardiac dynamics with clinical applicability.

The creation of new methodologies based on probability theory has made it possible to assess patients with arrhythmias, establishing their condition regarding mild abnormalities and tracking progression toward disease [7]. Previous methodologies developed with epidemiological studies showed a sensitivity of 100%, a specificity of 73.3%, and a kappa value of 0.86 in a cohort of 115 patients [30]. The methodology developed here replaces the parameters and sub-parameters of previous studies, offering a diagnostic tool that differentiates between normal, chronic, acute, and pacemaker dynamics based on probability ranges (1000–3000), simplifying Holter evaluation.

Acausal theories developed by statistical mechanics [31,32] and modern physics [8,9,10,33] demonstrate that statistical or epidemiological methods are not always required, as these theories involve causal studies of multiple factors. The new methodology developed here demonstrates the simplicity and objectivity of calculations based on probability theory, which avoids the influence of multiple factors and leads directly to results. The application of theories such as dynamic systems theory and box-counting theory has allowed the development of diagnostic tools [12]. This methodology was later improved with the analysis of 200 cases, differentiating between normal and acute cardiac dynamics and achieving statistical validation with 100% sensitivity and specificity [34]. An exponential chaotic law was developed to analyze and differentiate normal cardiac dynamics from those of patients with arrhythmias [14]. Continuing along this line of research, this fractal law was applied to the attractor occupation space, differentiating normal, chronic, and acute cases in a 16-h Holter exam, creating a diagnostic tool with clinical applications [15].

Based on the chaotic attractors developed in the new methodology [12], numerical attractors were created, allowing for the evaluation of regions within the attractor and proportions of entropy, thereby distinguishing normal, chronic, and acute dynamics. This research was validated with 800 Holter exams and clinical validation in 800 patients. The outcome of this investigation was the development of a diagnostic tool with clinical applications [35]. All these methodologies in dynamic systems were based on the principle of the loaded die. However, unlike previous approaches, the methodology developed here simplifies the discrimination of normal, chronic, acute, and pacemaker cardiac dynamics.

Other physical and mathematical investigations in cardiology have involved the Zipf–Mandelbrot law. Various applications of this law have been made in cardiology, including induction with 50 Holter exams, which, through a blind study, confirmed the law’s ability to differentiate normal cardiac dynamics from acute ones, achieving 100% sensitivity and specificity and a kappa coefficient of 1 [36]. Subsequently, the same methodology was applied to a study involving 70 Holter exams from both normal cases and patients in intensive care units. This research successfully differentiated cardiac dynamics, with clinical validation again confirming the methodology with 100% accuracy [37]. Finally, software was developed and tested through a blind study with 80 Holter exams, confirming that the complexity of cardiac dynamics diminished in acute diseases and was consistently higher in cases of normality [38]. These works on the Zipf–Mandelbrot law and cardiac dynamics do not clearly differentiate chronic states. In contrast, the methodology presented here successfully distinguishes all types of dynamics, including those involving pacemaker devices, making it a more comprehensive diagnostic tool.

Goldberger developed a concept of normality-disease based on dynamic systems [11], while Huikuri applied it in the study of mortality in acute myocardial infarction [39]. Other studies have shown that, although chaos is often associated with heart problems, not all irregularities indicate disease. In fact, it has been found that before cardiac arrest, the ECG may exhibit episodes of high regularity, suggesting that a lack of variability can also be problematic [40]. In this study, the first author proposes a theoretical, quantifiable, and predictive conception of normality/disease, where repetitions of heart rate frequencies and their occurrence ranges distinguish the fluctuations in frequency over time. This approach demonstrates an expansion in repetitions for chronic cases, which then sharply decreases in acute cases and even more so in dynamics involving pacemakers.

Research into heart rate variability has intensified, employing both statistical and nonlinear dynamics approaches. Most of these studies are descriptive of cardiac dynamics and are associated with the study of various pathologies [41]. Heart rate variability has been used as a predictive factor for coronary events, strokes, and sudden cardiac death [42]. Other studies have evaluated its use in developing training loads for athletes [43]. Typically, variability studies calculate and analyze statistical measures and populations [44], i.e., epidemiological metrics. In contrast, the methodology proposed here allows for direct predictions applicable to any specific clinical case, making it a more effective diagnostic tool for monitoring cardiac pathologies.

In the circadian rhythm, there are two clocks. One is an internal molecular clock [44], and the other is the physiological and general clock of the suprachiasmatic nucleus of the hypothalamus. These regulate circadian rhythms of heart rate, blood pressure, and many other physiological variables. In the morning [45], heart rate, platelet aggregation, blood pressure, sympathetic tone, and cortisol levels increase significantly [46]. These events have been associated with the majority of cardiac arrests occurring during these hours of the day. Additionally, studies have examined alterations in sleep patterns [47]. While a regular circadian rhythm is expected according to physiological homeostasis [48], research in dynamic systems reveals that normality exhibits chaos [49,50]. In this study, cardiac dynamics fluctuations were found to be based on probability distributions. These fluctuations are chaotically self-organized for each dynamic, demonstrating that variability and the circadian rhythm do not follow a fixed rhythm but rather exhibit self-organization inherent to calculable and predictable heart rate frequency fluctuations. The homeostatic concept of physiology and dynamic systems is surpassed here by the mathematical self-organization of dynamics. Furthermore, it develops a diagnostic tool based on the principle of the loaded die [16] and probability theory. This work not only reveals a new concept but also proposes a predictive theory applicable to cardiac dynamics and the human body. This study can quantify the “normal circadian rhythm”, the fluctuations in probabilities, and how the system’s inherent self-organization deteriorates as it progresses toward disease. In a normal case, rather than assuming regular rises and falls, the rhythm exhibits its own irregular mathematical pattern. As it evolves into chronic or acute conditions, this self-organization diminishes, and this loss can be quantified mathematically.

In the future, clinical applications will be developed with a larger number of Holter recordings, specifying clinically relevant diseases such as acute myocardial infarction and others, as well as studying the progression from a chronic to an acute dynamic for population prevention. Additionally, clinical application software will be developed to automate calculations and perform diagnostics. Finally, hardware will be designed to technologically implement the predictive methodology.

This work is based on the method of theoretical physics, employing an induction model grounded in the probabilities of frequencies over time. It quantifies dynamics and compares the essential characteristics of probability distributions across four fundamental cardiac dynamics: normal, chronic, acute, and pacemaker. Its clinical validation, combined with the “loaded die principle” [16], elevates cardiology to the level of theoretical physics. Here, predictions are specific to each particular case, as opposed to the population-based, statistical, and static nature of variability. Probability distributions, in contrast, are dynamic, predictive, and preventive, demonstrating that the presumed regularity of the circadian rhythm is a bias, replaced here by fluctuations in probability distributions.

Other methodologies in medicine using physical and mathematical theories have addressed diverse challenges. For instance, adverse drug reactions in elderly patients have been characterized using the Zipf–Mandelbrot law and probability theory, highlighting trends useful for pharmacovigilance [51]. Similarly, this law has been applied to develop a diagnostic methodology for fetal monitoring in 100 pregnant women [24]. Probabilistic approaches have also been used to predict epidemics, such as malaria, through recurrent probability [52] and probability combined with entropy [53], successfully forecasting the spatiotemporal spread of malaria across 800 municipalities in Colombia. Further applications include predicting the binding of plasmodium falciparum peptides to HLA class II peptides [54], diagnosing squamous epithelial cells in the cervix using fractal geometry, and later diagnosing cervical cytology [55]. Predictive methods have also been employed for forecasting traffic accident-related deaths in Colombia by 2020, using probabilistic random walks [56]. Another methodology, based on set theory, was developed to predict TCD4 lymphocyte counts in HIV patients [57]. In a different line of research, the theory of intrinsic mathematical harmony was used to evaluate the geometric self-organization of arteries at a supramolecular level [58].

A device was developed that applies the proportional entropy methodology [59] to 18 h, and by 2021, the methodology had been improved and patented as a clinical tool titled “Device and System for Cardiac Monitoring and Pathology Prediction” [60].

## 5. Conclusions

This is the first study to apply the theory of equiprobable probability to the ranges of heart rate occurrence, creating an induction model with four prototypical cases (normal, chronic, acute, and pacemaker) for which probabilities were calculated and the most frequent probabilities were summed.

## Figures and Tables

**Table 1:** Some cardiac anomalies by age.

No	Age	Diagnosis
PN	25	Study within normal limits
PC	36	Frequent monomorphic ventricular and supraventricular extrasystoles
PA	59	Acute myocardial infarction (AMI)
PMP	72	Unicameral VVIR pacemaker functioning normally
5	40	Intermittent left bundle branch block
6	36	Secondary ventricular failure
7	55	Dilated cardiomyopathy; high probability of pulmonary embolism (Wells Score)
8	29	Sinus rhythm and AV/intraventricular conduction
9	49	AAI pacemaker set to 50 bpm
10	50	Acute myocardial infarction (AMI)
11	60	Primary thrombophilia; cardiac resynchronization therapy user
12	77	Hypertension and cardiac arrhythmia with frequent precordial pain
13	44	Permanent unicameral VVI-R pacemaker
14	60	VVI-R pacemaker
15	68	VVI-R pacemaker functioning normally
16	70	Atrial fibrillation with two morphologies, intraventricular conduction
17	55	Sinus pauses with a maximum duration of 3 s
18	28	Atrial extrasystoles and atrial tachycardia
19	22	Study within normal limits
20	35	Study within normal limits
21	41	Study within normal limits
22	50	Study within normal limits
23	40	Study within normal limits
24	27	Study within normal limits
25	55	Study within normal limits

Conventional clinical diagnosis of Holter tests. PN: normal prototype; PC: chronic prototype; PA: acute prototype; PMP: pacemaker prototype.

**Table 2:** P1 1000 to 2000.

Normal Prototype		Chronic Prototype		Acute Prototype		Pacemaker Prototype	
Rep	Freq	Rep	Freq	Rep	Freq	Rep	Freq
1858	90	1982	85	1993	95	1539	75
1858	85	1938	70	1921	85	1536	65
1814	65	1938	75	1407	80	1120	70
1792	90	1934	80	1392	75	1115	60
1792	85	1659	90	1316	75		
1780	70	1534	90	1288	85		
1484	70	1498	85	1215	75		
1354	60	1484	90	1202	80		
1331	55	1473	70	1160	95		
1216	70	1472	85	1119	85		
1203	60	1449	95	1087	100		
		1419	95				
		1174	80				
		1171	95				
		1162	100				
		1161	90				
		1140	95				
		1122	100				
		1101	85				
		1086	100				
		1080	75				
		1065	90				
		1029	90				
Total	11	Total	23	Total	11	Total	4

**Table 3:** P1 2001 to 3000.

Normal Prototype		Chronic Prototype		Acute Prototype		Pacemaker Prototype	
Rep	Freq	Rep	Freq	Rep	Freq	Rep	Freq
2023	90	2524	80	2999	90	2412	65
		2496	75	2997	100	2397	60
		2427	75	2279	90		
		2426	80	2219	80		
		2184	75				
		2161	70				
		2124	70				
		2094	85				
		2084	80				
		2073	85				
Total	1	Total	10	Total	14	Total	2

**Table 4:** P1 1000 to 3000.

DX	Normal		Chronic		Acute		Pacemaker	
Ranges	Rep	Freq	Rep	Freq	Rep	Freq	Rep	Freq
P	0.45	1	0.43	0.6	0.54	0.75	0.5	1
2	0.45	0	0.4	0.54	0.54	0.75	0.55	1
3	0.38	0	0.42	0.59	0.52	0.75	0.6	1
4	0.4	0	0.39	0.66	0.5	0.75	0.59	1
5	0.39	1	0.38	0.69	0.5	0.75	0.58	1
6	0.41	0	0.35	0.55	0.55	0.5	0.5	1
7	0.4	1	0.48	0.7	0.56	0.52	0.5	1
8	0.42	1	0.36	0.7	0.6	0.46	0.53	1
9	0.44	1	0.38	0.59	0.58	0.48	0.58	1
10	0.39	0	0.4	0.69	0.59	0.5	0.57	1
11	0.45	1	0.4	0.55	0.6	0.7	0.54	1
12	0.45	1	0.41	0.66	0.6	0.72	0.52	1
13	0.45	1	0.43	0.7	0.51	0.75	0.6	1
14	0.45	1	0.42	0.59	0.53	0.75	0.6	1
15	0.45	0	0.45	0.54	0.6	0.69	0.6	1
16	0.46	0	0.48	0.54	0.5	0.62	0.55	1
17	0.46	1	0.48	0.63	0.5	0.62	0.6	1
18		1	0.48	0.62	0.6	0.46	0.58	1
19		0	0.35	0.68	0.52	0.46	0.52	1
20		1	0.35	0.64	0.55	0.55	0.56	1
21		0	0.48	0.54	0.55	0.46	0.51	1
MAX.	0.46	1	0.48	0.7	0.6	0.75	0.6	1
MIN.	0.35	0	0.35	0.54	0.5	0.46	0.5	1

**Table 5:** (**a**) Distributions and frequencies to normal and chronic. (**b**) Distributions and frequencies to acute and pacemaker.

(**a**)											
**Normal Prototype**						**Chronic Prototype**					
**P1 1000 to 2000**			**P2 2001 to 3000**			**P1 1000 to 2000**			**P2 2001 to 3000**		
**Rep**	**Freq**	**Prob**	**Rep**	**Freq**	**Prob**	**Rep**	**Freq**	**Prob**	**Rep**	**Freq**	**Prob**
55	1	0.09	55			55			55		
60	2	0.18	60			60			60		
65	1	0.09	65			65			65		
70	3	0.27	70			70	2	0.087	70	2	0.2
75			75			75	2	0.087	75	3	0.3
80			80			80	2	0.087	80	3	0.3
85	2	0.18	85			85	4	0.17	85	2	0.2
90	2	0.18	90	1	1	90	6	0.26	90		
95			95			95	4	0.17	95		
100			100			100	3	0.13	100		
(**b**)											
**Acute Prototype**						**Pacemaker Prototype**					
**P1 1000 to 2000**			**P2 2001 to 3000**			**P1 1000 to 2000**			**P2 2001 to 3000**		
**Rep**	**Freq**	**Prob**	**Rep**	**Freq**	**Prob**	**Rep**	**Freq**	**Prob**	**Rep**	**Freq**	**Prob**
55			55			55			55		
60			60			60	1	0.25	60	1	0.5
65			65			65	1	0.25	65	1	0.5
70			70			70	1	0.25	70		
75	3	0.27	75			75	1	0.25	75		
80	2	0.18	80	1	0.25	80			80		
85	3	0.27	85			85			85		
90			90	2	0.5	90			90		
95	2	0.18	95			95			95		
100	1	0.09	100	1	0.25	100			100		

## Data Availability

The original contributions presented in this study are included in the article. Further inquiries can be directed to the corresponding author.

## Abbreviations

The following abbreviations are used in this manuscript:

PN	normal prototype
PC	chronic prototype
PA	acute prototype
PMP	pacemaker prototype
FREQ	heart rate recorded by the test according to the repetitions within the ranges
PROT	prototype
REP	number of repetitions observed during the Holter test
DX	diagnosis
MAX	the highest result among all summations
MIN	the lowest result among all summations
RF	number of times the repetition occurred in the heart rate
PPROB	probability of the number of times a repetition occurred in the frequency relative to the total repetitions
